# Sequentially aerated membrane biofilm reactors for autotrophic nitrogen removal: microbial community composition and dynamics

**DOI:** 10.1111/1751-7915.12079

**Published:** 2013-10-01

**Authors:** Carles Pellicer-Nàcher, Stéphanie Franck, Arda Gülay, Maël Ruscalleda, Akihiko Terada, Waleed Abu Al-Soud, Martin Asser Hansen, Søren J Sørensen, Barth F Smets

**Affiliations:** 1 Department of Environmental Engineering, Technical University of DenmarkBuilding 113, Miljøvej, 2800, Kgs Lyngby, Denmark; 2 Laboratory of Chemical and Environmental Engineering (LEQUIA-UdG), Facultat de Ciències, Institute of the Environment, University of GironaCampus Montilivi s/n, E-17071, Girona, Catalonia, Spain; 3 Department of Chemical Engineering, Tokyo University of Agriculture & TechnologyNaka-cho 2-24-16, Koganei, 184-8588, Tokyo, Japan; 4 Department of Biology, Section for Microbiology, University of CopenhagenSølvgade 83H, 1307, Copenhagen K, Denmark

## Abstract

Membrane-aerated biofilm reactors performing autotrophic nitrogen removal can be successfully applied to treat concentrated nitrogen streams. However, their process performance is seriously hampered by the growth of nitrite oxidizing bacteria (NOB). In this work we document how sequential aeration can bring the rapid and long-term suppression of NOB and the onset of the activity of anaerobic ammonium oxidizing bacteria (AnAOB). Real-time quantitative polymerase chain reaction analyses confirmed that such shift in performance was mirrored by a change in population densities, with a very drastic reduction of the NOB *Nitrospira* and *Nitrobacter* and a 10-fold increase in AnAOB numbers. The study of biofilm sections with relevant 16S rRNA fluorescent probes revealed strongly stratified biofilm structures fostering aerobic ammonium oxidizing bacteria (AOB) in biofilm areas close to the membrane surface (rich in oxygen) and AnAOB in regions neighbouring the liquid phase. Both communities were separated by a transition region potentially populated by denitrifying heterotrophic bacteria. AOB and AnAOB bacterial groups were more abundant and diverse than NOB, and dominated by the *r*-strategists *Nitrosomonas europaea* and *Ca.* Brocadia anammoxidans, respectively. Taken together, the present work presents tools to better engineer, monitor and control the microbial communities that support robust, sustainable and efficient nitrogen removal.

## Introduction

The discovery of anaerobic ammonium oxidizing bacteria (AnAOB, aka anammox bacteria) two decades ago has launched a new phase in wastewater biotechnology. Many reactor concepts have been developed to take advantage of this functional group for the treatment of nitrogen (N)-rich waste streams. AnAOB can grow symbiotically with aerobic ammonium oxidizing bacteria (AOB) in biofilms with redox gradients, allowing the conversion of equimolar mixtures of ammonium (NH_4_^+^) and nitrite (NO_2_^−^) to nitrogen gas (N_2_) without the addition of organic carbon (Terada *et al*., 2011). In membrane-aerated biofilm reactors (MABRs) such redox gradient conditions can establish in a counter-diffusion mode, with oxygen (O_2_) entering the biofilm through the membrane–biofilm interface and NH_4_^+^ diffusing from the liquid phase into the biofilm at its surface. We have recently shown that this biofilm reactor configuration can effectively support autotrophic N removal from synthetic waste streams at a lower energy, spatial, and environmental footprint than is feasible by conventional (i.e. based on co-diffusion) biofilm technologies (Pellicer-Nàcher *et al*., 2010; Gilmore *et al*., 2013).

NO_2_^−^ is a central intermediate in autotrophic N conversions: it is the product of AOB and is a necessary substrate for AnAOB. Therefore, metabolically active nitrite oxidizing bacteria (NOB) are undesirable during autotrophic N removal, as the oxidation of NO_2_^−^ to nitrate (NO_3_^−^), catalysed by NOB, would hamper AnAOB activity. Several strategies have been explored to suppress or control NOB growth in suspended growth or co-diffusion biofilm systems such as operation at elevated pH, low dissolved oxygen (DO) concentration, or higher temperatures (Van Hulle *et al*., 2010). However, none of these procedures has proven efficient to suppress NOB activity in MABRs (Wang *et al*., 2009; Terada *et al*., 2010). In large part, this difficulty in outcompeting NOB may be due to the localization of NOB, this microbial guild, in MABR biofilms. NOB, when present, grow in the aerobic regions of the biofilm (inner biofilm regions due to O_2_ diffusion across the biofilm base) where DO and NO_2_^−^ concentrations are highest, and any changes applied to the liquid phase will have minimal effects. New approaches such as careful inoculum selection or implementation of cyclic aeration patterns have been explored and proven successful to moderate NOB activity in MABRs (Pellicer-Nàcher *et al*., 2010; Terada *et al*., 2010).

While the above studies have demonstrated the feasibility and identified suitable operational conditions for MABRs targeting autotrophic N removal (Pellicer-Nàcher *et al*., 2010; Gilmore *et al*., 2013), direct inspection of the microbial community structure and composition in the resulting MABR biofilms has been limited. Such inspection is not only necessary to assess the robustness of the process through the study of the diversity of the established microbial community, but also to deepen the understanding about how biofilms can be engineered for a certain operational purpose by applying selected operational strategies. In addition, information from direct biofilm inspection can be used to support or modify biofilm process models, where community compositions are easily predicted but rarely verified (Terada *et al*., 2013; Lackner *et al*., 2008).

Here, we present the first exhaustive characterization study of the structure and composition of microbial biofilms that support autotrophic N removal in MABRs (Pellicer-Nàcher *et al*., 2010). We are especially keen to verify whether the imposed redox stratification results in the predicted ecological stratification of the involved functional groups, whether suppression of NOB and stimulation of AnAOB activity by sequential aeration is mirrored by the abundances of NOB and AnAOB, whether operational and reactor conditions have resulted in a more or less diverse set of functional guilds, and whether heterotrophic bacteria (HB) coexist in this autotroph-dominated community. Hence, we used a complementary set of molecular and microscopic tools to identify, quantify and assess the microbial diversity and structure of biofilms in a long-term operated MABR run under O_2_ limitation and treating a synthetic NH_4_^+^ rich influent.

## Results and discussion

### Microbial dynamics during reactor operation

**Fig. 1:**
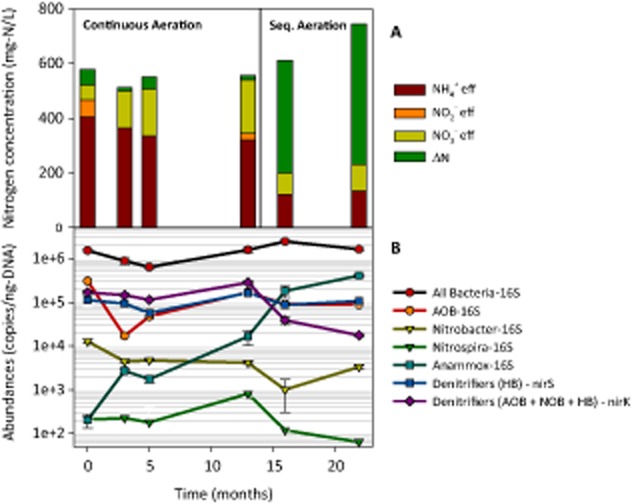
Reactor performance and microbial community abundances during reactor operation. Month 0: reactor start-up after AnAOB inoculation. 23 months: reactor shutdown.A. Averaged reactor performance during biomass sampling periods. Concentrations of NH_4_^+^, NO_2_^−^, NO_3_^−^ and the N denitrified/assimilated -ΔN- are stacked, yielding the NH_4_^+^ concentration in the influent.B. Population dynamics measured by real-time quantitative polymerase chain reaction (qPCR).

From inoculation to month 13, the reactor was operated in continuous aeration mode. The O_2_ to NH_4_^+^ loading ratio was controlled at the optimal value for complete NH_4_^+^ conversion to N_2_ via the nitritation-anammox pathway (Terada *et al*., 2013). Notwithstanding the imposed O_2_ limited operation, most of the NH_4_^+^ was simply converted to NO_3_^−^, resulting in limited N removal (Fig. 1A). From month 13 onward, O_2_ was supplied in cycles of aeration/non-aeration, dramatically affecting reactor performance. Initially, strong NO_2_^−^ accumulation was observed, followed by an increase in AnAOB activity and nearly maximal N removal (70%). In addition, at this point, minimal nitrous oxide (N_2_O) emissions were observed (Pellicer-Nàcher *et al*., 2010).

Imposition of the sequential aeration regime caused clear shifts in the microbial community, as suggested by the results obtained from real-time quantitative polymerase chain reactions (qPCR) using relevant primers (Fig. 1B): the abundance of *Nitrobacter* spp. decreased by an order of magnitude. *Nitrobacter* spp. are considered *r*-strategist NOB (Schramm, 2003), whose presence is correlated with poor nitritation efficiencies (Terada *et al*., 2010). Both 16S rRNA gene (Fig. 1B) and *nxrA* (Fig. 1; in the Supporting Information, Fig. S1) targeted NOB quantifications were consistent. The gene copy numbers of *Nitrobacter* spp. increased by the end of the experiment (still under sequential aeration), but they did not negatively affect reactor performance (Fig. 1A). The abundance of the *Nitrospira* spp. was also severely affected by the onset of the sequential aeration: it decreased and dropped to the quantification limit until the end of the experiment. These data suggest that the *Nitrospira* NOB, despite their lower abundance vis-à-vis *Nitrobacter* NOB, were responsible for the conversion of NO_2_^−^ to NO_3_^−^ during the antecedent continuous aeration phase. This NOB genus is known to thrive in environments with low NO_2_^−^ concentrations (*K*-strategist, Schramm, 2003). The overall reduction in NOB abundance was mirrored by a significant increase in AnAOB numbers, as reflected by 16S rRNA gene (Fig. 1B) and hzo-targeted quantifications (Fig. S1). The exact reasons explaining the observed suppression of the NOB upon onset of the sequential aeration regime are unknown, but we hypothesize that AOB are less affected by the feast/famine conditions associated with cyclic aeration (Geets *et al*., 2006) and display a higher affinity for O_2_ at low concentrations than NOB (Blackburne *et al*., 2008), which would allow AOB to outcompete NOB for the transiently available limiting O_2_. AOB are known to release hydroxylamine and NO during O_2_ transients, compounds which may have also inhibited NOB (Schmidt *et al*., 2003; Noophan *et al*., 2004; Kostera *et al*., 2008). The increased NO_2_^−^ availability (due to NOB suppression), and the postulated transiently available NO may also have enhanced NO_2_^−^ uptake by AnAOB upon the onset of cyclic aeration (Jetten *et al*., 2009; Kartal *et al*., 2010). Although NO_2_^−^ concentrations (in the bulk phase) attained values as high as 200 mg-N l^−1^, they did not prevent the stimulation of AnAOB activity, in contrast with earlier studies that reported NO_2_^−^ values as low as 28 mg-N l^−1^ to negatively affect their activity (van der Graaf *et al*., 1996).

The density of denitrifying bacteria was approximated by quantifying the abundance of the *nirK* and *nirS* genes; *nirK* and *nirS* encode NO_2_^−^ reductase enzymes in both heterotrophic denitrifying bacteria (*nirS* and *nirK*) or nitrifiers (*nirK,* Braker *et al*., 1998; Casciotti and Ward, 2001; Heylen *et al*., 2006). Onset of cyclic aeration caused a significant decrease in *nirK* abundance, while *nirS* remained relatively constant. Because AOB abundance remained fairly constant, this trend suggests a shift in the heterotrophic denitrifying guild, becoming more *nirS* abundant as AnAOB density and activity increased. The decrease in NOB, also known to contain *nirK* in their genome (Schreiber *et al*., 2012), could have contributed as well to the observed drop. Prior work revealed that N_2_O emissions from our and other MABRs were very low when AnAOB activity was high (∼0.015% and < 0.001% N-N_2_O/ N-load during the aerated and non-aerated phase of an aeration cycle, respectively, Pellicer-Nàcher *et al*., 2010), but can be very high when AnAOB activity is low (2–11% N-N_2_O/ N-load; Gilmore *et al*., 2013). Because N_2_O emissions are, in part, caused by NO_2_^−^ reductase (Zumft, 1997), it will be interesting to verify whether the dramatic drop in *nirK* abundance directly correlates with reduction in N_2_O emissions.

### Microbial community composition and architecture

**Fig. 2:**
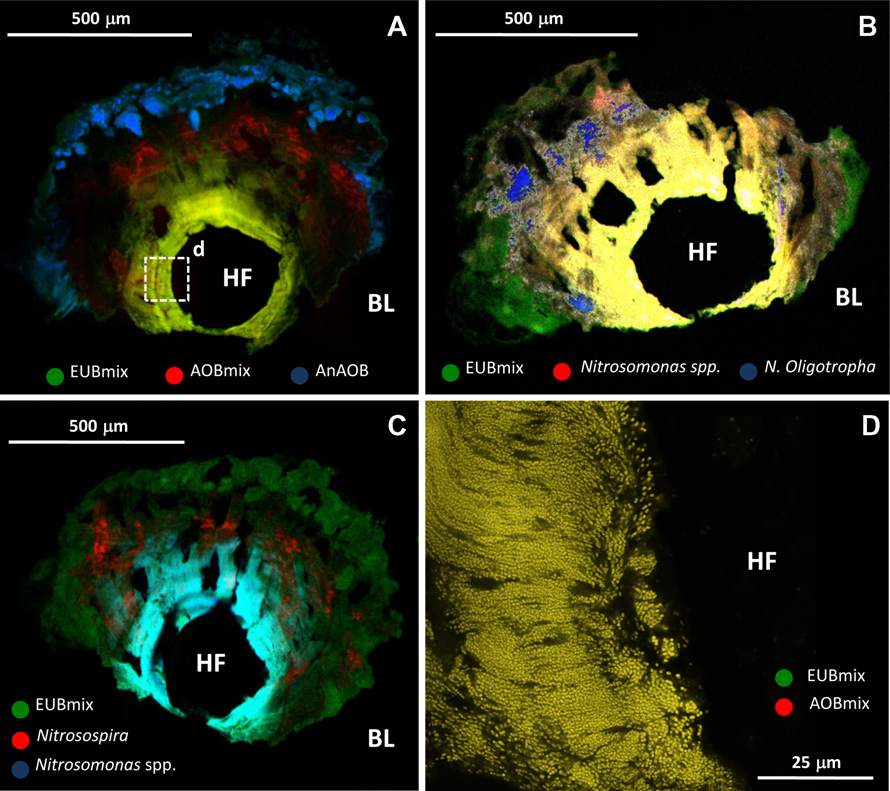
Biofilm cross-sections hybridized with fluorescent probes targeting AOB and AnAOB. HF and BL stand for hollow fiber and bulk liquid respectively.A. Biofilm cross-section hybridized with probes targeting All Bacteria (EUBmix), AOB and AnAOB.B. Biofilm section hybridized with probes targeting All Bacteria (EUBmix), halophilic and halotolerant *Nitrosomonas* spp and *N. oligotropha*.C. Biofilm section hybridized with probes targeting All Bacteria (EUBmix), halophilic and halotolerant *Nitrosomonas* spp. and *Nitrosospira* spp.D. Detail of Fig. 2A at the membrane–biofilm interface (d).

After 23 months of operation (630 days), the developed biofilm exhibited a very pronounced radial stratification of the microbial community (Fig. 2A). The biofilm region adjacent to the hollow fibre membrane was clearly dominated by AOB, as indicated by the simultaneous signal from EUB – all bacteria – and AOB targeting probes (Table 1, combination 1). The thickness of the observed AOB layer ranged from 110 to 170 μm across all analysed samples, comparable to the O_2_ penetration measured with microsensors during reactor operation here and in other nitritating MABRs (Fig. S2, Terada *et al*., 2010). Moreover, since the DO concentration in the bulk liquid was close to the detection limit of the DO probe used (Pellicer-Nàcher *et al*., 2010), it could be concluded that the AOB group consumed most of the O_2_ transferred from the membrane, and mediated the partial conversion of the NH_4_^+^ diffusing from the bulk liquid to NO_2_^−^. High magnification micrographs in this area revealed rod-shaped cells in very compact strata around the hollow fibre membranes (Fig. 2D). The average biofilm porosities calculated here were 0.36 ± 0.07, half of what is considered normal in co-diffusion biofilm systems (Zhang and Bishop, 1994). This packed and ordered structure of AOB is very different from the cauliflower-shaped clusters observed in other biofilm systems (Okabe and Kamagata, 2010), which may be caused from the pressure of upper biofilm strata onto the biofilm base, high growth velocities or the high competition for O_2_ and space in this region. Isolated cauliflower-shaped clusters could only be seen in biofilm regions distant from the membrane where DO concentrations were lower.

**Table 1:** Probe combinations used in the study (after Loy *et al*., 2007; Okabe and Kamagata, 2010). Probe sequences and hybridization conditions for each probe are available in Table S1

		Fluorophore		
		
No.	Experimental Purpose	FLUO (green)	Cy3 (red)	Cy5 (blue)
1.	Verify spatial distribution of AOB and AnAOB	EUBmix	Nso190-Nmo218-Cluster 6a192^a^	Amx820
2.	Examine abundance of *Nitrosospira* spp. vs halophilic and halotolerant *Nitrosomonas* spp. (AOB)	EUBmix	Nsv443	NEU^a^
3.	Examine abundance of *N. oligotropha* vs halophilic and halotolerant *Nitrosomonas* spp. (AOB)	EUBmix	NEU^a^	Nmo218-Cluster 6a192^a^
4.	Examine abundance of *Candidatus* Kuenenia spp. vs *Candidatus* Brocadia spp. (AnAOB)	EUBmix	Kst157	Ban162
5.	Examine abundance of *Nitrobacter* spp. vs *Nitrospira* spp. (NOB)	EUBmix	NIT3^a^	Ntspa662^a^

a Probe requires a competitor

The use of probes with higher phylogenetic resolution (Table 1, combinations 2 and 3, Fig. 2B and C), indicated that halophilic and halotolerant *Nitrosomonas* spp. were the dominant AOB in the system. Halophilic and halotolerant *Nitrosomonas* spp. are known to outcompete other AOB species in environments with high substrate availability (Okabe and Kamagata, 2010). The high NH_4_^+^ concentrations expected across the whole biofilm thickness (bulk concentrations ranged from 100 to 400 mg-N l^−1^) may hamper the ability of *Nitrosospira* spp. to thrive even in biofilm areas with lower DO concentrations, as previously reported (Schramm *et al*., 2000). Our observation is in agreement with the work by Terada *et al*. (2010), who noted that MABR biofilms populated by *Nitrosomonas* spp. rather than *Nitrosospira* spp. supported higher NO_2_^−^ production. Halophilic and halotolerant *Nitrosomonas* spp. are typically the most abundant AOB in co-diffusion biofilms performing autotrophic N removal (Sliekers *et al*., 2003; Vázquez-Padín *et al*., 2010; Liu *et al*., 2012).

Microcolonies of the *N. oligotropha* lineage (Table 1, probe combination 3, Fig. 1B) were occasionally observed and randomly distributed within aerobic biofilm regions. Although *N. oligotropha* is expected in environments of low substrate availability, *N. oligotropha* and *N. europaea* can coexist in aerobic regions of nitrifying biofilms when operated under dynamic aeration conditions (Gieseke *et al*., 2001). In another MABR biofilm performing autotrophic N removal under continuous aeration *N. oligotropha* and halophilic and halotolerant *Nitrosomonas* spp. were also identified as the most abundant AOB (Gilmore *et al*., 2013). In that study however, *N. oligotropha* signals were observed in anaerobic strata parallel to the membrane surface, suggesting cell maintenance without growth, as *N. oligotropha* is, among other AOB, able to maintain its ribosome content even under famine conditions (Gieseke *et al*., 2001).

**Fig. 3:**
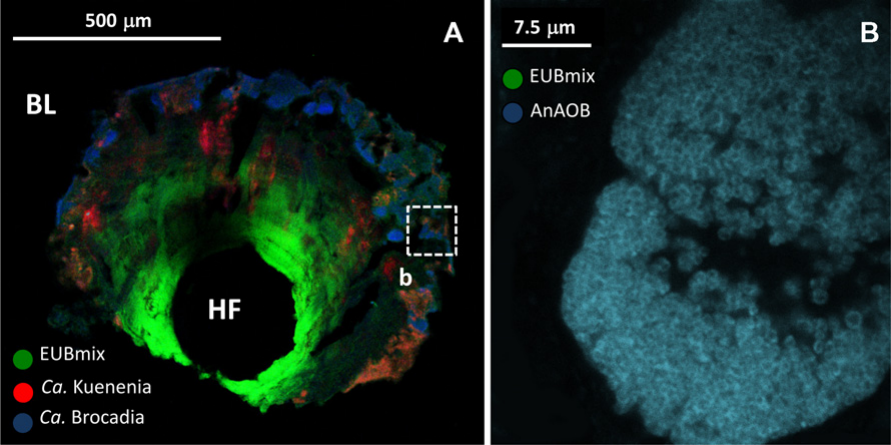
Biofilm cross-sections hybridized with fluorescent probes targeting AnAOB. HF and BL stand for hollow fiber and bulk liquid respectively.A. Biofilm cross-section hybridized with probes targeting All Bacteria (EUBmix), *Ca.* Brocadia, and *Ca.* Kuenenia.B. Detail of Fig. 3-A in regions neighbouring the bulk liquid (b).

AnAOB-positive signals were exclusively found close to the biofilm top, at least 300 μm away from the membrane surface (Fig. 2A). Microsensor measurements confirmed that DO was almost absent in this region (Fig. S2), while high NH_4_^+^ and NO_2_^−^ concentrations were expected, given their concentrations in the bulk liquid (Fig. 1A). AnAOB microcolonies were spearhead- or oval-shaped with round edges. High magnification micrographs (Fig. 3B) revealed doughnut-shaped cellular morphology, characteristic of AnAOB (rRNA distributes around the central anammoxome organelle, Kuenen, 2008).

Although it is rare to find multiple AnAOB species in bioreactors performing autotrophic N removal (Hu *et al*., 2010; Park *et al*., 2010a), both *Ca.* Brocadia anammoxidans (dark cyan) and *Ca.* Kuenenia stuttgartiensis (light orange) were observed here. However, *Brocadia* signals were clearly more abundant (Fig. 3A, Table 1, combination 4). These two AnAOB have different postulated growth preferences: *Ca.* Brocadia seems to thrive in environments with high substrate availability (*r*-strategist), while *Ca.* Kuenenia finds its niche in environments with nutrient scarcity (*K*-strategist) (van der Star *et al*., 2008). Microcolonies of both AnAOB lineages do not appear to be stratified within our biofilms. This observation might be due to the changing NH_4_^+^ and NO_2_^−^ concentrations during sequential aeration. In a continuously aerated MABR performing autotrophic N removal exclusively the *Ca.* Brocadia AnAOB lineage was observed (Gilmore *et al*., 2013).

**Fig. 4:**
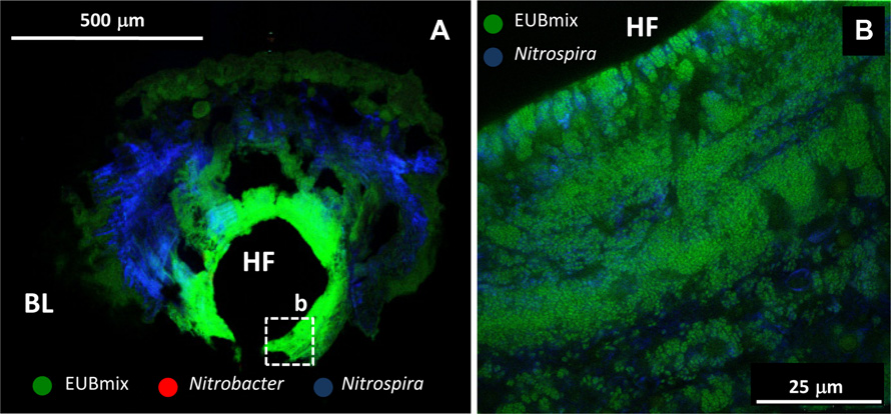
Biofilm cross-sections hybridized with fluorescent probes targeting NOB. HF and BL stand for hollow fiber and bulk liquid respectively.A. Biofilm section hybridized with probes targeting All Bacteria (EUBmix), *Nitrobacter* and *Nitrospira*.B. Detail of Fig. 4A in a region close to the aeration membrane (b).

Overall reactor mass balance calculations suggested that, although substantial N removal was observed (5.5 g-N m^−2^ day^−1^), approximately 30% of the NO_3_^−^ production was due to residual NOB activity (0.3 g-N m^−2^ day^−1^), while the remainder had been synthesized by AnAOB (0.7 g-N m^−2^ day^−1^, Pellicer-Nàcher *et al*., 2010). Here, NOB could still be detected in the biofilm (Table 1, combination 5, Fig. 4), even though they were clearly outnumbered by AOB in the aerobic biofilm regions. NOB in these biofilms were exposed to dual competitive pressure caused by fast growing AOB (consuming most O_2_) and AnAOBs (consuming the generated NO_2_^−^; Terada *et al*., 2010; Gilmore *et al*., 2013). Only *Nitrospira*, a *K*-strategist NOB, was observed in the biofilm after fluorescent in-situ hybridization (FISH) inspection (Fig. 4A), while *Nitrobacter*, detectable via qPCR analyses, was not observed, suggesting their lower activity. In well-functioning co-diffusion autotrophic N removal biofilms, both NOB types have been found (Sliekers *et al*., 2002; Park *et al*., 2010a).

While *Nitrospira* signals appeared most abundant distant from the biofilm base, scattered *Nitrospira*-positive cells were also detected in the aerobic regions close to the biofilm–membrane interface (Fig. 4B). This observation is in contrast with a previous MABR study, which suggested that DO concentrations at the membrane–biofilm interface above 63 μM result in a shift in the NOB community from *Nitrospira* to *Nitrobacter*, leading to a reduction in nitritation efficiencies (Downing and Nerenberg, 2008). In our system, DO concentrations at the membrane–biofilm interface were as high as 300 μM (Fig. S2), and yet no *Nitrobacter* signals were observed. High nitritation turnover is therefore possible in MABRs under intensive O_2_ loading conditions if a cyclic aeration pattern is imposed.

**Fig. 5:**
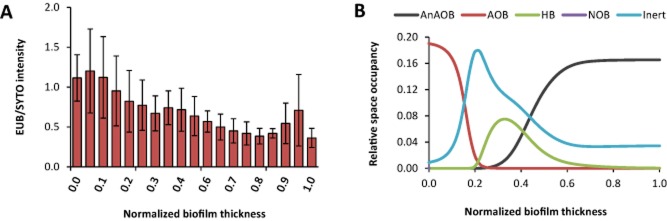
Observed and predicted occurrence of metabolically active biofilm regions. Normalized biofilm thickness = 1 (biofilm top). Normalized biofilm thickness = 0 (membrane–biofilm interface).A. Averaged relative activity profile with biofilm depth (n = 4). Standard deviations represented with error bars in each column.B. Relative space occupancy profile for the considered bacterial communities calculated by mathematical modelling in a previous study describing heterotrophic activity in MABR biofilms for autotrophic N removal (Lackner *et al*., 2008).

Between the aerobic (AOB-signal rich) and anaerobic (AnAOB-signal rich) biofilm regions, a transitional zone existed where no conclusive phylogenetic assignments could be made based on the employed FISH probes. A non-sense probe (non-EUB) was successfully applied at the first stages of the study to rule out any autofluorescence or unspecific probe binding in this region. Additional analyses of metabolic activity were made by normalizing the cellular ribosomal content (using EUBmix signal as proxy) to the total cellular genomic content (using SYTO 60 signal as proxy) across the biofilm. This analysis yielded three tentative peaks (Fig. 5A); the peaks at the biofilm base (0) and top (1) were indicative of the described AOB and AnAOB regions. A third additional peak in the centre of the biofilm (normalized biofilm thickness of 0.4) might indicate the presence of metabolically active cells in this transition region (Fig. 5A), even though the increase in signal intensity observed was not statistically significant, as could be concluded from the ANOVA test performed. Application of double-labelled oligonucleotide probes (DOPE probes, Stoecker *et al*., 2010) did not improve the assignments (results not shown). Earlier modelling efforts in MABRs for autotrophic N removal predicted the accumulation of HB and large amounts of bacterial debris in this transition zone (Fig. 5B, Lackner *et al*., 2008). Together with the qPCR results (showing abundant *nirS* numbers, indicative of heterotrophic denitrifiers), this suggests an anoxic transition zone populated by a heterotrophic denitrifying community. In addition, the non-specific and low probe signals observed suggest that these regions contained biomass debris and non-viable cells, consistent with the described model predictions.

### Microbial community abundances

Quantitative image analysis of the presented FISH results revealed that AOB and AnAOB accounted for 53 ± 13% and 38 ± 6% of the biofilm, with 9 ± 8% of the EUB signals not accounted for by either AOB or AnAOB (Table 1, combination 1). A separate analysis indicated a highly variable NOB fraction constituting 11 ± 10% of all detected EUB signals (Table 1, combination 5). These AOB, NOB and AnAOB fractions are consistent with those observed by FISH analysis in co-diffusion biofilms performing autotrophic N removal (Liu *et al*., 2012).

These qFISH results compare with those obtained by qPCR, for which AOB, NOB and AnAOB fractions were estimated at about 5, 0.2 and 25% of the total community population (considering that each cell contained a single copy of the genes targeted by qPCR primers, Table 2). Differences in sampling procedure may have impacted considerably the AOB and NOB results obtained by qPCR. While the entire biofilm is probed and imaged during FISH analysis (both biofilm and membrane were cryosectioned), only the biomass that could be scraped off the membranes was quantified by qPCR. As nitrifiers were preferentially present adjacent to the aeration membrane, AOB and NOB underestimation by qPCR was likely. Additionally, DNA extraction methods are known to display different yields in different types of bacterial species, which may bias substantially later qPCR analyses (Juretschko *et al*., 2002; Foesel *et al*., 2008). The targeted functional genes may be present in multiple copies per cell in the different biocatalysts studied, which could also lead to wrong estimates of the community fractions by qPCR calculated here (Pei *et al*., 2010). Finally, small differences could also be explained by the fact that, oligonucleotide probes (for FISH) and primers (for qPCR) do not necessary detect exactly the same phylogenetic clades (Hallin *et al*., 2005).

**Table 2:** Relative abundance of functional microbial guilds after 630 days of operation assessed via different molecular techniques

	Pyrosequencing^a^	qPCR	FISH
AOB	2.70 ± 0.87%	5.4 ± 0.2%	53.7 ± 13.2%
NOB	0.57 ± 0.12%	0.2 ± 0.2%	11.4 ± 10%
AnOB	60.00 ± 3.42%	25.0 ± 12.1%	38.2 ± 5.8

a Taxonomy index of each functional guild was given in supplementary document (Table S1).

The presented results, however, seem to converge in the fact that AOB and AnAOB are the most abundant microbial guilds. Model calibration and process operation can greatly benefit from the dual detection approach presented here. While FISH and microelectrode results would give information on the position and relative abundances of the functional microbial communities (Schramm *et al*., 2000), qPCR performed on representative biomass samples would allow for routine observation measurements on the microbial dynamics of the system. These observations could alert about unwanted changes in the microbial community supporting the process and suggest the need of imposing process control to correct deviations from the desired set point (Park *et al*., 2010a).

### Diversity of community fractions assessed by deep sequencing

**Fig. 6:**
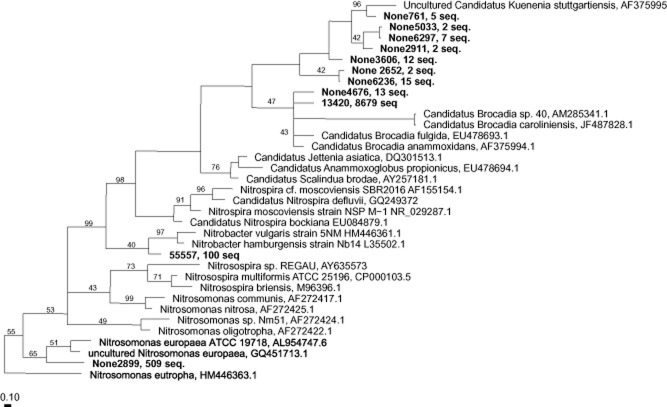
Phylogenetic tree of identified AOB, NOB and AnAOB sequences, and reference strains. Sequences from 16S rRNA libraries were assembled in OTUs based on 97% similarity. Distance matrices were computed with Jukes-Cantor method, and phylogenetic trees were rendered based on neighbour joining with bootstrap replication. Numbers at the branch nodes indicate bootstrap values over 40%. Number of identified sequences in each OTU is listed. OTUs with singular sequences are not shown. Full tree is available in Figure S4.

Microbial diversity of relevant functional guilds (AOB, NOB and AnAOB) was revealed by pyrosequencing the V3-V4 region of amplified community 16S rDNA. After implementation of quality control measures and denoising, 19 962 sequences were obtained from triplicate samples, which could be binned in 439 operational taxonomic units based on 97% phylogenetic similarity (OTU_0.03_). A total of 15, 2 and 6 OTUs_0.03_ could be assigned to AnAOB, NOB and AOB respectively (Fig. 6). All three sequence libraries shared 117 OTUs_0.03_ but were distinct due to abundant unshared genotypes (Fig. S3).

A remarkable diversity was detected within the AnAOB guild containing 15 OTUs_0.03,_ while sampling depth may not yet have revealed the complete diversity. Sequences of both the *Kuenenia* and *Brocadia* lineages were detected. While species richness in both lineages was the same (8 each), *Brocadia* sequences were significantly more abundant (290:1), consistent with FISH observations. Sequences affiliated to the aerobic ammonium oxidizer *Nitrosomonas europaea* (509 seq.) and the NO_2_^−^ oxidizer *Nitrobacter hamburgensis* (100 seq.) were the dominant nitrifying bacteria in the studied MABR biofilms (Fig. 6). While the diversity of AOB was relatively high, species different to *N. europaea* represented only minor fractions of the AOB guild (most having a single sequence for each OTU_0.03_, occurring in only one of the replicates, Fig. S4). Higher microbial diversity is normally reported in systems with dynamic operation conditions (Rowan *et al*., 2003).

Heterotrophic OTUs_0.03_ (41) were found in all samples despite the absence of organic carbon in the synthetic feed. The most abundant HB sequences were mainly assigned to the *Xanthomonadaceae* (γ-Proteobacteria, 550 seq.) and *Clostridiaceae* (Firmicutes, 356 seq.) families. Our results are in agreement with the findings of a study applying microautoradiography combined with FISH (MAR-FISH) to unravel the patterns in the cross-feeding of microbial products originating from nitrifier decay to HB (Okabe *et al*., 2005). In that study *Chloroflexi* and *Cytophaga-Flavobacterium* cluster cells were responsible for the degradation of slow biodegradable material (e.g. cells). Furthermore, members of the α- and γ-*Proteobacteria* (e.g. *Xanthomonadaceae,* typical in low carbon environments) metabolized other low-molecular weight organic substrates. Members of the *Clostridiaceae* family were not detected by their approach (gram positive bacterial require special fixation protocols), but, like *Xanthomonadaceae,* they are also known to degrade complex organic substrates and use NO_3_^−^ and NO_2_^−^ as electron acceptor in anaerobic environments (Finkmann *et al*., 2000; Wüst *et al*., 2011). CARD FISH may further assist in the identification of the organisms present in this intermediate region.

The fractions of AOB, NOB and AnAOB were identified based on the number of sequences detected by pyrosequencing. The results were further compared to those obtained by qPCR and FISH (Table 2). The abundance results obtained by pyrosequencing confirm the reduced NOB abundance with respect to AOB and AnAOB. The quantification of AOB and *Nitrospira* spp. was also affected with respect to qFISH. As previously indicated, such a divergence in results can arise by the difficulty in detaching the aerobic biofilm regions from the membrane or by a lower DNA extraction efficiency of the proposed treatment for these microbial species. The differences observed among PCR-based techniques could be related to the different amplification efficiency of universal primers (used in pyrosequencing) compared with family- and genus-specific primers (used in qPCR). Indeed, universal primers enhance the detection of highly abundant taxa but disfavour species represented by lower fractions (Gonzalez *et al*., 2012).

## Conclusion

Imposition of sequential aeration was successful in the rapid suppression of NOB activity and stimulating and maintaining AnAOB activity in the MABR, at very high effective O_2_ loadings (4-14g-O_2_ m^−2^ day^−1^). qPCR-based analysis revealed the following most remarkable shifts in the biofilm composition: a strong and moderate decrease in, respectively, the *nirK*, *Nitrospira* and *Nitrobacter* 16S rRNA gene abundance, and a strong increase in the AnAOB 16S rRNA gene abundance.

FISH analysis of the biofilms, after long-term sequential aeration, confirmed radial microbial stratification, with AOB at very high cellular densities in the O_2_-rich areas close to the membrane surface, and AnAOB located close to the bulk liquid, separated by a transition region potentially harbouring denitrifying HB supported by decay products. Extant diversity assessed by using phylogenetically defined FISH probes revealed that halophilic and halotolerant *Nitrosomonas* spp., *Ca.* Brocadia anammoxidans and *Nitrospira* spp. were the most abundant lineages within the AOB, NOB and AnAOB guilds respectively. AOB and AnAOB constituted the most abundant community fractions, outnumbering NOB. Deep sequencing of the mature biofilm showed that the AOB guild was dominated by a single *N. europaea* OTU and confirmed a diverse AnAOB guild containing one most abundant *Brocadia* spp. OTU. Overall, our multipronged analysis confirmed that sequential aeration regimes can be used to successfully to engineer a microbial community to attain high-rate autotrophic N removal.

## Experimental procedures

### Samples

Samples were obtained from a MABR performing stable N removal at 5.5 g-N m^−2^ day^−1^ via the nitritation-anammox pathway. This reactor housed 10 membrane bundles, each containing 128 30 cm long hollow-fibres (Model MHF3504, polyethylene/polyurethane, Mitsubishi Rayon, Tokyo, Japan). The reactor was inoculated in two steps with biomass from nitrifying (day −30) and AnAOB enrichment cultures (day 0), and was fed a synthetic NH_4_^+^-rich influent (100–500 mg-N l^−1^), while air was passed through the fibre lumens (2.5-40 kPa and 2.5–60 l min^−1^). After 390 days of operation the reactor was aerated in sequential cycles comprising aerated and non-aerated periods (Pellicer-Nàcher *et al*., 2010). On days 90, 150, 390 and 480, biofilm samples were detached and removed from the reactor by inserting a Pasteur pipette through the installed sampling ports and aspiring biomass from several fibre bundles at several heights. In these samples, biofilm structure could not be preserved. More comprehensive and structurally intact biofilm samples were obtained sacrificially at the end of the reactor run (day 630).

### FISH analysis

Biofilm samples were collected after 630 days of operation by cutting one fibre at three different locations along its length (lower, middle and upper). Specimens containing both biofilm and membrane were fixed in 4% paraformaldehyde, embedded in OCT compound (Sakura Finetek Europe, Zoeterwoude, the Netherlands), frozen at −21°C, cut in 20 μm-thick sections by using a microtome, and mounted on gelatine-coated slides. Sectioned samples were dehydrated and sequentially probed with Fluorescein (FLUO), Cy3- and Cy5- tagged 16S rRNA probes (Sigma Aldrich, St Louis, MO, USA, Table 1), following procedures described elsewhere (Terada *et al*., 2010). Biofilm structure was further studied by staining the prepared sections with a general fluorescent nucleic acid stain following manufacturer's specifications (SYTO 60, Invitrogen, Carlsbad, CA, USA).

Hybridized and stained sections were inspected with a confocal laser-scanning microscope (CLSM, Leica TCS SP5, Leica Microsystems, Wetzlar, Germany) equipped with an Ar laser (488 nm), and two HeNe lasers (543 and 633 nm). Gain and pinhole settings were tuned for each registered image in order to allow unsaturated exposure values for all detected pixels. At least five micrographs obtained from specimens hybridized with probe combinations 1 and 5 (Table 1) were used to quantify the abundance of AOB, AnAOB and NOB using the image analysis software Daime (Daims *et al*., 2006). Images of SYTO stained biofilms were analysed with Leica AS AF Lite (Leica) and ImagePro (MediaCybernetics, Des Moines, IA, USA) software to determine biofilm porosities and probe intensity profiles along biofilm depth.

### DNA extraction

DNA was extracted from 0.5 g of biofilm mass (dry weight) using the MP FastDNA™ SPIN Kit (MP Biomedicals, Solon, OH, USA) according to manufacturer's instructions. The quality of the extracted genomic DNA was checked by measuring its 260/280 nm absorbance ratio with a NanoDrop (ThermoFisher Scientific, Waltham, MA, USA) and was later stored at −20°C in Tris-EDTA buffer until further processing.

### High-resolution tag-based 16S rRNA sequence library

Biofilm DNA sampled on day 630 was amplified with Phusion (Pfu) DNA Polymerase (ThermoFisher Scientific) and the 16S rDNA universal primers PRK341F (5′-C CTAYGGGRBGCAACAG-3′) and PRK806R (5′-GG ACTACNNGGGTATCTAAT-3′) (Yu *et al*., 2005) with 25 annealing and elongation cycles. Detailed description of the PCR amplification conditions have been described before (Masoud *et al*., 2011; Sundberg *et al*., 2013). Amplified DNA was purified using the QIAquick PCR purification kit (Qiagen, Vento, the Netherlands) according to the manufacturer's protocol. In a second 15-cycle PCR round, barcodes and tags were added. All 16S rDNA fragments comprising the V3 and V4 hypervariable regions were pyrosequenced using a 454 FLX Titanium sequencer (Roche, Penzberg, Germany). Detailed description of the pyrosequencing preparation process can be found elsewhere (Farnelid *et al*., 2011).

### Bioinformatic analyses

All raw 16S rDNA amplicons were processed and classified using the QIIME (http://qiime.org/index.html) software package (Caporaso *et al*., 2010b). Chimera checking and denoising were performed with the software Ampliconnoise (Quince *et al*. 2011). Retrieved sequences were clustered at 97% evolutionary similarity and aligned against the Greengenes reference set (DeSantis *et al*., 2006) using the Pynast algorithm (Caporaso *et al*., 2010a). Taxonomy index of each functional guild is given in Table S2. Phylogenetic trees were created in ARB using library sequences of interest together with selected representatives from a small subunit (SSU) reference library (SSU Ref. Nr. 111 Silva). Statistical calculations and phylogenetic comparisons were carried out using R software (R Development Core Team, 2012).

### Quantitative PCR (qPCR)

Biofilm DNA sampled and extracted throughout the whole operational period (days 0, 90, 150, 390, 480 and 630) was subject to qPCR to determine the numbers of AOB, NOB, AnAOB and denitrifying bacteria, based on appropriate 16S rRNA targets or functional genes (Table 3). Details on the procedure can be found elsewhere (Pellicer-Nàcher *et al*., 2010; Terada *et al*., 2010).

**Table 3:** Primers and conditions used for the quantification of bacterial numbers by qPCR

Primers	Target Organism	Target molecule	Sequence (5′-3′)	Reference
1055f 1392r	All Bacteria	Eubacterial 16S rRNA gene	ATG GCT GTC GTC AGC T ACG GGC GGT GTG TAC	Lane, 1991; Ferris *et al*., 1996
CTO189fa/b CTO189fc RT1r	β-proteobacterial AOBs	16S RNA gene	GGA GRA AAG CAG GGG ATC G GGA GGA AAG TAG GGG ATC G CGT CCT CTC AGA CCA RCT ACT G	Kowalchuk *et al*., 1997; Hermansson and Lindgren, 2001
FGPS872f 1269r	*Nitrobacter* NOB	16S RNA gene	CTA AAA CTC AAA GGA ATT GA TTT TTT GAG ATT TGC TAG	Degrange and Bardin, 1995
Nspra675f 746r	*Nitrospira* NOB	16S RNA gene	GCG GTG AAA TGC GTA GAK ATC G TCA GCG TCA GRW AYG TTC CAG AG	Graham *et al*., 2007
F1370f1 F2843r2	*Nitrobacter*/*Nitrococcus* NOB	nxrA gene	CAG ACC GAC GTG TGC GAA AG TCC ACA AGG AAC GGA AGG TC	Poly *et al*., 2008; Wertz *et al*., 2008
Amx809f Amx1066r	AnAOB	16S RNA gene	GCC GTA AAC GAT GGG CAC T ATG GGC ACT MRG TAG AGG GGT TT	Tsushima *et al*., 2007
hzoqf hzoqr	AnAOB	Hzo gene	CAT GGT CAA TTG AAA GRC CAC C GCC ATC GAC ATA CCC ATA CTS	Park *et al*., 2010b
cd3aF R3cd	Denitrifying bacteria	nirS gene	AAC GYS AAG GAR ACS GG GAS TTC GGR TGS GTC TTS AYG AA	Throbäck *et al*., 2004
F1aCu R3Cu	Denitrifying bacteria	nirK gene	ATC ATG GT(C/G) CTG CCG CG GCC TCG ATC AG(A/G) TTG TGG TT	Hallin and Lindgren, 1999

### Microelectrode measurements

Oxygen Clark-type microsensors were constructed to measure DO concentrations within the biofilm. Preparation, calibration and microsensor measurement were performed as previously described (Revsbech, 1989; Pellicer-Nàcher *et al*., 2010).

## Supporting information

**Table S1.** Probes used for the detection of target organisms by in-situ fluorescent hybridization.

**Table S2.** Index of related taxonomy for each functional guild.

**Fig. S1.** Reactor performance and microbial community abundances during reactor operation (qPCR performed with primers targeting functional genes).

**Fig. S2.** Typical O2-mircoprofiles during aeration periods within an aeration cycle.

**Fig. S3.** Rarefaction curves of denoised sequences and shared and unique OTUs between triplicate samples.

**Fig. S4.** Phylogenic tree constructed with all the identified AOB, NOB and AnAOB sequences.
