# Supplementary material for: Sequentially aerated membrane biofilm reactors for autotrophic nitrogen removal: microbial community composition and dynamics
Source: Microb Biotechnol. 2013 Oct 1;7(1):32–43. doi: 10.1111/1751-7915.12079 (PMC3896934; doi:10.1111/1751-7915.12079)
Supplement: Supplementary file 1 [file mbt20007-0032-SD1.docx]

*Supporting information:*

Sequentially aerated membrane biofilm reactors for autotrophic nitrogen removal: Microbial community composition and dynamics

Running title: Microbial study of MABR biofilms for autotrophic N removal

**C. Pellicer-Nàcher^1^, S. Franck^1^, A. Gülay^1^, M. Ruscalleda^2^, A. Terada^3^, W. A. Al-Soud^4^, M. A. Hansen^4^, S. J. Sørensen^4^, and B. F. Smets^1*^**

*Considered for publication in*

*Microbial Biotechnology*

^1^ Department of Environmental Engineering, Technical University of Denmark, Building 113, Miljøvej, 2800 Kgs Lyngby, Denmark. **Phone:** +45 45251600. **FAX:** +45 45932850. **e-mail:** capn@env.dtu.dk, s072796@student.dtu.dk, argl@env.dtu.dk, bfsm@env.dtu.dk*

^2^ Laboratory of Chemical and Environmental Engineering (LEQUIA-UdG), Facultat de Ciències, Institute of the Environment, University of Girona, Campus Montilivi s/n, E-17071, Girona, Catalonia. **Phone:** +34 972419542. **e-mail:** mael@lequia.udg.cat

^3^ Department of Chemical Engineering, Tokyo University of Agriculture & Technology, Naka-cho 2-24-16, Koganei, 184-8588 Tokyo, Japan. **Phone:** +81 423887069. **FAX:** +81 423887693. **e-mail:** akte@cc.tuat.ac.jp

^4^ Department of Biology, University of Copenhagen, Sølvgade 83H, 1307 Copenhagen K, Denmark. **Phone:** +45 35323710. **FAX:** +45 35322128. **e-mail**: maahansen@bio.ku.dk, w.abualsoud@bio.ku.dk, sjs@bio.ku.dk

*Corresponding author

**Table S1.** Probes used for the detection of target organisms by in-situ fluorescent hybridizations (built from (Loy et al., 2007))

| **Probe name** | **Target** | **Probe sequence (5’-3’)** | **FA (%)** | **Reference** |
| --- | --- | --- | --- | --- |
| EUB338 | Most Bacteria | GCT GCC TCC CGT AGG AGT | 35 | (Amann et al., 1990) |
| EUB338 II | *Planctomycetales* | GCA GCC ACC CGT AGG TGT | 35 | (Daims et al., 1999) |
| EUB338 III | *Verrucomicrobiles* | GCT GCC ACC CGT AGG TGT | 35 | (Daims et al., 1999) |
| Nso190 | β-proteobacterial AOB | CGA TCC CCT GCT TTT CTC C | 35 | (Mobarry et al., 1996) |
| Nmo218 | *N. oligotropha* lineage | CGG CCG CTC CAA AAG CAT | 35 | (Gieseke et al., 2001) |
| Cluster6a192 | *N. oligotropha* lineage | CTT TCG ATC CCC TAC TTT CC  CTT TCG ATC CCC TGC TTT CC | 35 | (Adamczyk et al., 2003) |
| Nsv443 | Most *Nitrosospira* spp. | CCG TGA CCG TTT CGT TCC | 30 | (Mobarry et al., 1996) |
| NEU | Most halophilic and halotolerant *Nitrosomonas* spp. | CCC CTC TGC TGC ACT CTA  TTC CAT CCC CCT CTG CCG | 40 | (Wagner et al., 1995) |
| Amx820 | Most AnAOB | AAA ACC CCT CTA CTT AGT GCC C | 40 | (Schmid et al., 2001) |
| Kst157 | *Candidatus* Kuenenia stuttgartiensis | GTT CCG ATT GCT CGA AAC | 25 | (Schmid et al., 2001) |
| Ban162 | *Candidatus* Brocadia anammoxidans | CGG TAG CCC CAA TTG CTT | 40 | (Schmid et al., 2001) |
| NIT3 | *Nitrobacter* spp. | CCT GTG CTC CAT GCT CCG  CCT GTG CTC CAG GCT CCG | 40 | (Wagner et al., 1995) |
| Ntspa662 | Genus *Nitrospira* | GGA ATT CCG CGC TCC TCT  GGA ATT CCG CTC TCC TCT | 35 | (Daims et al., 2001) |

**Table S2.** Index of related taxonomy for each functional guild in pyrosequencing analysis

|  | **Domain** | **Phylum** | **Class** | **Order** | **Family** |
| --- | --- | --- | --- | --- | --- |
| AOB | Bacteria | *Proteobacteria* | Beta-*proteobacteria* | *Nitrosomonadales* | *Nitrosomonadaceae* |
|  | Bacteria | *Proteobacteria* | Gamma-*proteobacteria* | *Chromatiales* | *Nitrosococcus* |
| NOB | Bacteria | *Proteobacteria* | Alpha-*proteobacteria* | *Rhizobiales* | *Bradyrhizobiaceae* |
|  | Bacteria | *Nitrospirae* | *Nitrospira* | *Nitrospirales* | *Nitrospiraceae* |
|  | Bacteria | *Proteobacteria* | Gamma-*proteobacteria* | *Chromatiales* | *Ectothiorhodospiraceae* |
|  | Bacteria | *Proteobacteria* | Delta-*proteobacteria* | *Desulfobacterales* | *Nitrospinaceae* |
| AnAOB | Bacteria | *Plantomycetes* | *Kueneniae* | *Kueneniales* | *Kueneniaceae* |
| Heterotrophs | Bacteria | *Firmicutes* | *Clostridia* | *Clostridiales* | *Clostridiaceae* |
|  | Bacteria | *Acidobacteria* | *Solibacteres* | *Solicacterales* | *Solibacteraceae* |
|  | Bacteria | *Firmicutes* | *Clostridia* | *Clostridiales* | *Veillonellaceae* |
|  | Bacteria | *Actinobacteria* | *Actinobacteria* | *Actinomycetales* | *Microbacteriaceae* |
|  | Bacteria | *Firmicutes* | *Clostridia* | *Clostridiales* | *Lachnospiraceae* |
|  | Bacteria | *Firmicutes* | *Clostridia* | *Clostridiales* | F. XIII. *Incertae Sedis* |
|  | Bacteria | *Proteobacteria* | Gamma-*proteobacteria* | *Xanthomoadales* | *Xanthomoadaceae* |
|  | Bacteria | *Proteobacteria* | Beta-*proteobacteria* | *Burkholderiales* | *Burkholderiaceae* |
|  | Bacteria | *Proteobacteria* | Beta-*proteobacteria* | *Rhodocyclales* | *Rhodocyclaceae* |
|  | Bacteria | *Proteobacteria* | Beta-*proteobacteria* | *Burkholderiales* | *Comamonadaceae* |

**Figure S1.** Reactor performance and microbial community abundances during reactor operation (qPCR performed with primers targeting functional genes). Month 0: Reactor start-up after AnAOB inoculation. 23 months: reactor shutdown. A. Averaged reactor performance during biomass sampling periods. Concentrations of NH_4_^+^, NO_2_^-^, NO_3_^-^ and the N denitrified/assimilated -ΔN- are stacked, yielding the NH_4_^+^ concentration in the influent. B. Population dynamics measured by qPCR using primers targeting functional genes.

**Figure S2.** Typical O_2_-mircoprofiles during aeration periods within an aeration cycle. The dashed line represents the membrane-biofilm interface. Figure created by Frank Schreiber.

**A**

**B**

**Figure S3.** A. Rarefaction curves of denoised sequence library of samples from studied MABR based on Chao species richness estimator. B. Shared and unique OTUs between triplicate samples from a 630 day operated MABR


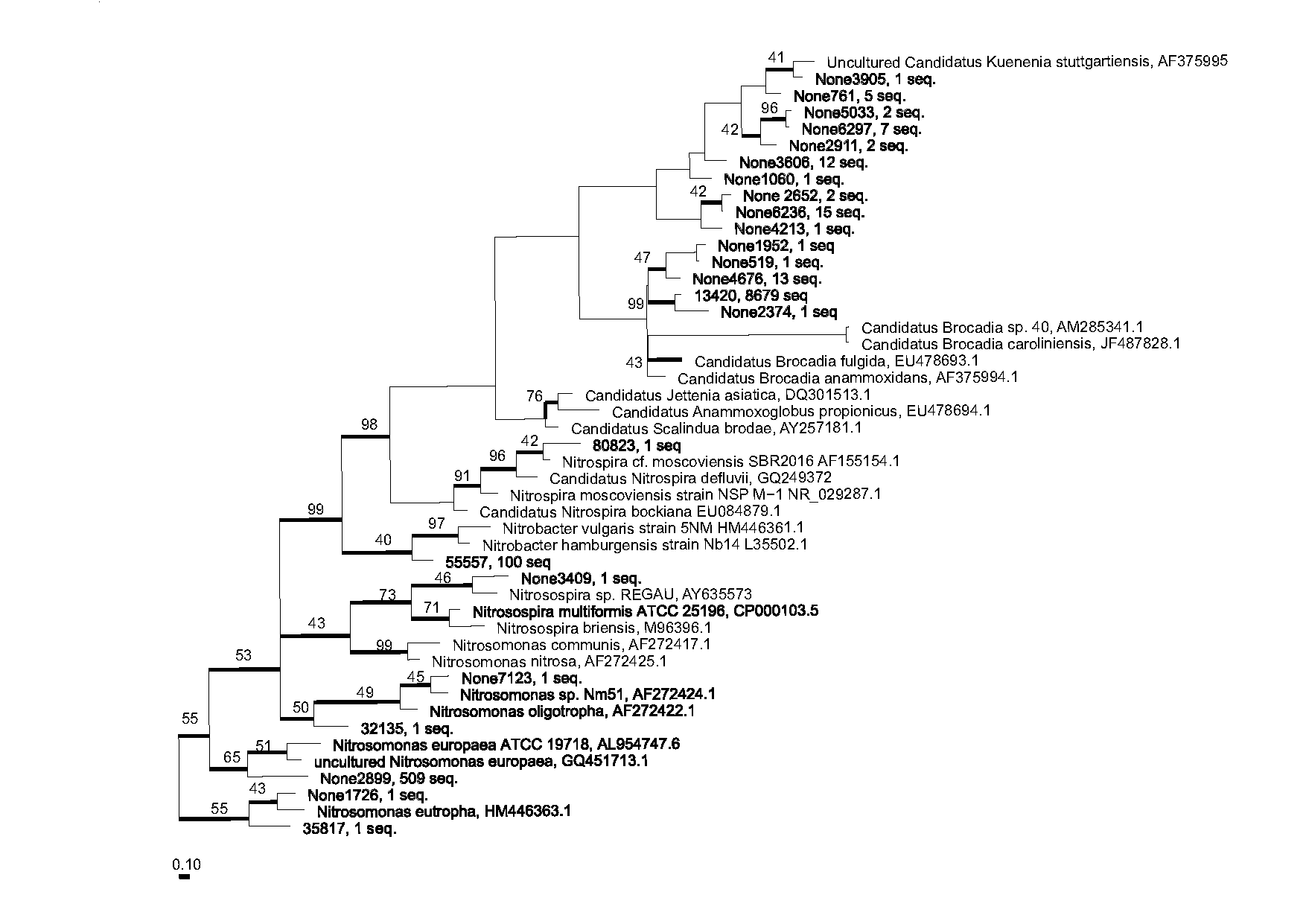


**Figure S4.** Phylogenetic tree of identified AOB, NOB and AnAOB sequences, and reference strains. Sequences from 16S rRNA libraries were assembled in OTUs based on 97% similarity. Distance matrices were computed with Jukes-Cantor method, and phylogenetic trees were rendered based on neighbour joining with bootstrap replication. Numbers at the branch nodes indicate bootstrap values over 40%. Number of identified sequences in each OTU is listed

# References

Adamczyk J., Hesselsoe M., Iversen N., Horn M., Lehner A., Nielsen P.H., et al. 2003. The isotope array, a new tool that employs substrate-mediated labeling of rRNA for determination of microbial community structure and function. *Appl. Environ. Microbiol.* **69**: 6875–6887.

Amann R.I., Binder B.J., Olson R.J., Chisholm S.W., Devereux R., and Stahl D.A. 1990. Combination of 16S rRNA-targeted oligonucleotide probes with flow cytometry for analyzing mixed microbial populations. *Appl. Environ. Microbiol.* **56**: 1919–1925.

Daims H., Brühl A., Amann R., Schleifer K.-H., and Wagner M. 1999. The domain-specific probe EUB338 is insufficient for the detection of all bacteria: Development and evaluation of a more comprehensive probe set. *Systematic and Applied Microbiology* **22**: 434–444.

Daims H., Nielsen J.L., Nielsen P.H., Schleifer K.-H., and Wagner M. 2001. In situ characterization of *Nitrospira*-like nitrite-oxidizing bacteria active in wastewater treatment plants. *Appl. Environ. Microbiol.* **67**: 5273–5284.

Gieseke A., Purkhold U., Wagner M., Amann R., and Schramm A. 2001. Community structure and activity dynamics of nitrifying bacteria in a phosphate-removing biofilm. *Appl. Environ. Microbiol.* **67**: 1351–1362.

Loy A., Maixner F., Wagner M., and Horn M. 2007. ProbeBase—an online resource for rRNA-targeted oligonucleotide probes: new features 2007. *Nucleic Acids Res.* **35**: D800–D804.

Mobarry B.K., Wagner M., Urbain V., Rittmann B.E., and Stahl D.A. 1996. Phylogenetic probes for analyzing abundance and spatial organization of nitrifying bacteria. *Appl. Environ. Microbiol.* **62**: 2156–2162.

Schmid M., Schmitz-Esser S., Jetten M., and Wagner M. 2001. 16S-23S rDNA intergenic spacer and 23S rDNA of anaerobic ammonium-oxidizing bacteria: implications for phylogeny and in situ detection. *Environ. Microbiol.* **3**: 450–459.

Wagner M., Rath G., Amann R., Koops H.-P., and Schleifer K.-H. 1995. In situ identification of ammonia-oxidizing bacteria. *Systematic and Applied Microbiology* **18**: 251–264.
